# NDM Production as a Dominant Feature in Carbapenem-Resistant Enterobacteriaceae Isolates from a Tertiary Care Hospital

**DOI:** 10.3390/antibiotics11010048

**Published:** 2021-12-31

**Authors:** Fakhur Uddin, Syed Hadi Imam, Saeed Khan, Taseer Ahmed Khan, Zulfiqar Ahmed, Muhammad Sohail, Ashraf Y. Elnaggar, Ahmed M. Fallatah, Zeinhom M. El-Bahy

**Affiliations:** 1 Jinnah Postgraduate Medical Center (JPMC), Department of Microbiology, Basic Medical Sciences Institute (BMSI), Karachi 75510, Pakistan; fakharindhar@hotmail.com; 2 Basildon University Hospital Essex, Basildon SS16 5NL, UK; dr_hadiimam@hotmail.com; 3 Department of Pathology, Dow University of Health Sciences (DUHS), Karachi 74200, Pakistan; saeed.khan@duhs.edu.pk; 4 Department of Physiology, University of Karachi, Karachi 75270, Pakistan; takhan@uok.edu.pk (T.A.K.); zuahmed@uok.edu.pk (Z.A.); 5 Department of Microbiology, University of Karachi, Karachi 75270, Pakistan; 6 Department of Food Nutrition Science, College of Science, Taif University, P.O. Box 11099, Taif 21944, Saudi Arabia; aynaggar@tu.edu.sa; 7 Department of Chemistry, College of Science, Taif University, P.O. Box 11099, Taif 21944, Saudi Arabia; a.fallatah@tu.edu.sa; 8 Department of Chemistry, Faculty of Science, Al-Azhar University, Nasir City, Cairo 11884, Egypt; zeinelbahy@azhar.edu.eg

**Keywords:** carbapenem-resistant Enterobacteriaceae, metallo-β-lactamases, NDM producers

## Abstract

The worldwide spread and increasing prevalence of carbapenem-resistant Enterobacteriaceae (CRE) is of utmost concern and a problem for public health. This resistance is mainly conferred by carbapenemase production. Such strains are a potential source of outbreaks in healthcare settings and are associated with high rates of morbidity and mortality. In this study, we aimed to determine the dominance of NDM-producing Enterobacteriaceae at a teaching hospital in Karachi. A total of 238 Enterobacteriaceae isolates were collected from patients admitted to Jinnah Postgraduate Medical Centre (Unit 4) in Karachi, Pakistan, a tertiary care hospital. Phenotypic and genotypic methods were used for detection of metallo-β-lactamase. Out of 238 isolates, 52 (21.8%) were CRE and 50 isolates were carbapenemase producers, as determined by the CARBA NP test; two isolates were found negative for carbapenemase production by CARB NP and PCR. Four carbapenemase-producing isolates phenotypically appeared negative for metallo-β-lactamase (MBL). Of the 52 CRE isolates, 46 (88.46%) were *bla*_NDM_ positive. Most of the NDM producers were *Klebsiella pneumoniae*, followed by *Enterobacter cloacae* and *Escherichia coli*. In all the NDM-positive isolates, the *bla*_NDM_ gene was found on plasmid. These isolates were found negative for the VIM and IPM MBLs. All the CRE and carbapenem-sensitive isolates were sensitive to colistin. It is concluded that the NDM is the main resistance mechanism against carbapenems and is dominant in this region.

## 1. Introduction

Many Enterobacteriaceae species are the pathogens involved in hospital-associated and community-acquired infections, especially in urinary and respiratory tracts, the blood stream, and intra-abdominal and surgical sites [1]. The most commonly encountered pathogens of the family Enterobacteriaceae are *Escherichia coli*, *Klebsiella pneumoniae, Proteus, Salmonella, Shigella* and *Enterobacter* spp. These genera are reportedly very susceptible to carbapenems [2]. Hence, carbapenems are considered as a good option to treat the infections caused by extended-spectrum β-lactamase (ESBL) producing strains and other multidrug-resistant (MDR) bacteria [3,4]. Owing to the emergence (up to 32%) of carbapenem-resistant Enterobacteriaceae (CRE) and their inclusion in the list of priority pathogens, they have received attention globally [4,5,6,7,8,9]. CRE strains can also spread resistance markers by horizontal transfer to other strains in hospitals and augment problems in healthcare sectors [4]. In the case of bloodstream infections, CRE significantly increase the mortality rate up to 65.4% in comparison to carbapenemase-susceptible Enterobacteriaceae pathogens [8]. CRE strains have a common mechanism of resistance against carbapenem antibiotics by producing carbapenemases [4].

Many types and subtypes of carbapenemases (*bla*_IMP_, *bla*_VIM_, *bla*_SIM_, *bla*_SPM_, *bla*_GIM_, *bla*_KPC_, *bla*_SME_) have been recognized among Enterobacteriaceae; the arrival of NDM-1 is the ‘final straw’ in this increasing antimicrobial resistance problem [10]. Rapid and easy determination of carbapenemases in Enterobacteriaceae is needed for effective clinical practices and infection control measures. Different phenotypic approaches are employed for the diagnosis of CPE, including the modified Hodge test (MHT), Blue-Carba test and Rapidec CARBA NP test. The Rapidec CARBA NP test is a biochemical assay with a good sensitivity and specificity, but it cannot differentiate among types of carbapenemases [7,11]. This test is also recommended by the Clinical and Laboratory Standards Institute (CLSI) [12]. Likewise, another inhibitory test, EDTA with imipenem or meropenem, is a phenotypic test for the detection of metallo-β-lactamases in Enterobacteriaceae. Both tests are cost-effective and are feasible for the screening of carbapenemases and metallo-β-lactamases prior to performing costly and sophisticated genotypic tests in low-income countries.

This cross-sectional, single-center study was conducted on the admitted patients in the medical unit of Jinnah Postgraduate Medical Centre in Karachi, Pakistan. The different clinical specimens were collected according to the site of infection. Traditional microbiological techniques and some cost-effective assays were used for the detection of the carbapenemases, with specific emphasis on metallo-β-lactamases. The goal of this research was three-fold: to monitor antimicrobial resistance among the members of Enterobacteriaceae, to detect the presence of CRE and to study the prevalence of metallo-β-lactamases producing Enterobacteriaceae by phenotypic and genotypic methods.

## 2. Results

**Figure 1:**
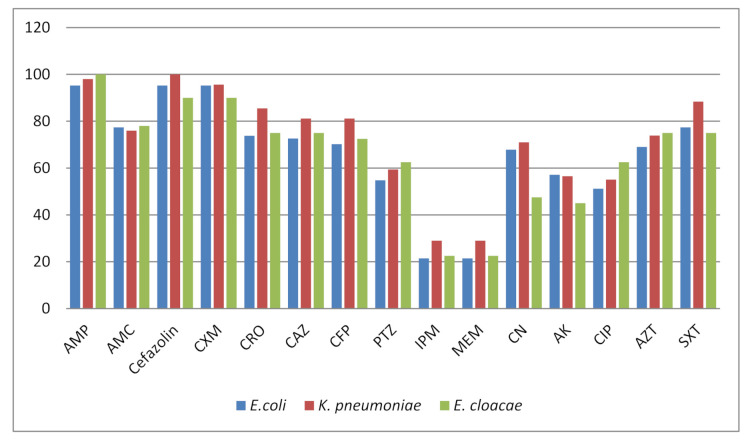
Resistance pattern of major isolated pathogens *Escherichia coli*, *Klebsiella pneumoniae* and *Enterobacter cloacae*. AMP: ampicillin, AMC: amoxicillin/clavulanic acid, CXM: cefuroxime, CRO: ceftriaxone, CAZ: ceftazidime, CFP: Cefepime, PTZ: piperacillin/tazobatam, IPM: imipenem, MEM: meropenem, CN: gentamicin, AK: amikacin, CIP: ciprofloxacin, AZT: aztreonam, SXT: sulfamethoxazole-trimethoprim.

Among 238 Enterobacteriaceae isolates, *E. cloacae* (40; 16.80%), *K. pneumonia* (69; 28.99%) and *E. coli* (84; 35.29%) were the most common pathogens (Table 1). *Klebsiella aerogenes, K. oxytoca, P. mirabilis, P. vulgaris* and *S. typhi* were isolated less frequently. Out of 238 isolates, 52 (21.84%) were CRE. The carbapenem resistance was higher in *Klebsiella aerogenes* (3; 23.1%), *K. pneumoniae* (20; 28.9%), *E. cloacae* (9; 22.5%) and *E. coli* (18; 21.4%) in comparison to other species of Enterobacteriaceae (Table 2). The isolates of different species are small in number, and for the validation of these results, the large scale studies are required. The resistance against the ampicillin, cefazolin and cefuroxime was 90–100%. A large number of isolates (46.2% to 83.3%) exhibited resistance against β-lactamase inhibitors and cephalosporin. The resistance to aminoglycosides was 45–71% (Figure 1), and all the isolates were susceptible to colistin, except the *Proteus* spp. having intrinsic resistance. Out the 52 CRE isolates, 46 (88.5%) found carbapenemase producers by the phenotypic colorimetric assay, Rapedic CARBA NP. The metallo-β-lactamase detection was determined by the phenotypic inhibitor based EDTA+ IMP and MEM discs. A total of 43 (82.7%) showed metallo-β-lactamase producers using this method. For the confirmation by the PCR assay, 41 (78.8%) were positive for *bla*_NDM_. The *bla*_VIM_ and *bla*_IMP_ could not be detected in any isolate, and out of 52 CRE, 11(21%) isolates were negative for *bla*_NDM_*, bla*_VIM_ and *bla*_IMP_. The *bla*_NDM_ positive isolates were analyzed for the plasmid extraction, and PCR was performed; all the isolates showed the presence of *bla*_NDM_ on plasmids. The conjugation results revealed that *bla*_NDM_ was transferred to *E. coli* J53 recipient successfully from 38 *bla*_NDM_ positive isolates, and 03 *E. coli* isolates failed to show the transmissibility by conjugation on several attempts.

## 3. Discussion

The incidence of infection by the carbapenem-resistant Enterobacteriaceae (CRE) is increasing worldwide and poses a threat to public health and a challenge for physicians [2]. Among CREs, *K. pneumoniae* is a leading pathogen, followed by *E. coli* and *E. cloacae* [13]. The common mode for carbapenem resistance in Enterobacteriaceae is the production of carbapenemases, particularly New Delhi metallo-β-lactamase (NDM-1), on the Indian subcontinent [14]. The carbapenem resistance and carbapenemase production in Enterobacteriaceae vary geographically with respect to prevalence, pathogen and type of carbapenemase, so it is necessary to continuously monitor the presence and prevalence of these bugs [15]. Although, *K. pneumoniae* Carbapenemase-2 (KPC-2) has been found dominantly in China, NDM is the most frequently detected (71.4%) metallo-β-lactamase in Hunan among CRE isolates, as reported by Chinese network for CRE surveillance [13]. The prevalence of carbapenem resistance in Enterobacteriaceae isolates was 21.84% in the present study. The resistance to other classes of β-lactams, including cephalosporins, penicillins and aztreonam, was higher (61–100%) in comparison to ciprofloxacin and aminoglycosides. This higher resistance to penicillins and cephalosporins may be attributed to the higher prevalence (60–80%) of ESBLs, modification in the outer membrane porins or AmpC overexpression or other mechanisms as deciphered for the isolates from Asia [16].

Comparing carbapenem resistance among the members of Enterobacteriaceae, most of the *K. pneumoniae* isolates appeared resistant, followed by *Enterobacter* spp. and *E. coli*. In the majority of CRE isolates (88.5%), carbapenemase production was the main mode of resistance to carbapenems by the phenotypic Rapidec CARBA NP test. This is a colorimetric, phenotypic test that is easy to perform without any special requirements. A simple color change can be read by the technician, so it does not require highly skilled or specially trained staff. This provides rapid identification of carbapenemase-producing strains within 30 min to 2 h, at a lower cost in comparison to the molecular assays [17]. This is helpful for the screening of carbapenemase-producing isolates, especially in low- and middle-income countries, including Pakistan, where molecular assays are not very common. The prevalence of metallo-β-lactamases was higher (82.69%) in CRE by phenotypic assay in the present study. These results were in accordance with the PCR results; therefore, this cost-effective technique may be used to screen metallo-β-lactamases in CRE isolates and provide information regarding the selection of therapeutic options.

NDM production is the main mode of resistance against the carbapenems in enterobactericeae, as found in this study. NDM was initially reported in *K. pneumoniae* and *E. coli* strains isolated from a Swedish patient, having history of seeking medical care in New Delhi, India, in 2009. It has since been spread all over the world and has been detected in different species of Enterobacteriaceae as well as in other Gram-negative bacilli. Unlike in North America and Europe, NDM, IMP and VIM are the most common carbapenemases in CRE in Southeast Asia and were similarly observed in the present work in Karachi, Pakistan. The previous data revealed that the NDM is endemic in Pakistan, Bangladesh and India, while KPCs are endemic in Colombia, Brazil, Argentina and USA [7,13]. Surveillance reports from India and neighboring countries summarized that, in Enterobacteriaceae, the most predominant carbapenemase is NDM [7]. These findings coincide with the present work. The higher prevalence of NDM producers in this study revealed that the NDM-harboring isolates are dominant in this region. A previous study from Pakistan reported that the major carbapenemase among the carbapenemase-producing Enterobacteriaceae is NDM [18].

The results of the plasmid DNA extraction and conjugation assay indicated that the *bla*_NDM_ is plasmid mediated, although a more sensitive assay needs to be performed to confirm this conclusion. Similar findings were reported in an earlier report [15]. In the present research work, the *bla*_VIM_ and *bla*_IPM_ could not be detected in the CRE isolates. However, these are reported in the members of Enterobacteriaceae isolates in the regions and health care settings where *bla*_VIM_ and *bla*_IPM_ carrying *P. aeruginosa* and other glucose non-fermenter Gram-negative bacilli are common [19,20]. In the present work six CRE isolates were negative for carbapenemase production by the Rapidec CARBA NP test, and 11 isolates were negative for *bla*_NDM_, *bla*_IPM_ and *bla*_VIM_ by PCR. These isolates may carry any AmpC, KPC or OXA carbapenemses in combination with other carbapenem-resistant mechanisms, including overexpression of efflux pumps and the decreased permeability to carbapenems.

## 4. Materials and Methods

### 4.1. Setting

The study was conducted in the Department of Microbiology, Basic Medical Sciences Institute (BMSI) and Medical Unit-4 of Jinnah Postgraduate Medical Centre (JPMC) Karachi, Pakistan, in collaboration with the department of Microbiology and the department of Physiology, University of Karachi.

### 4.2. Sample Size and Collection

A total of 238 Enterobacteriaceae isolates were included in this study. The clinical specimens were collected from January to June 2018 from medical Unit-4 (Department of Medicine and Critical Care), which comprises a general ward and an ICU. The samples were collected from the urine patients suspecting of having urinary tract infections, from respiratory secretions including endotracheal tubes, tracheal aspirates and sputum for respiratory tract infections, and from blood in the case of septicemia. One isolate per patient was included, and the repeated samples of the same patient were excluded in the present study according to the adjusted criteria.

*Inclusion criteria:* During this period all the admitted patients presenting urinary tract infection, hospital acquired pneumonia and bacteremia were included.

*Exclusion criteria*: Isolates other than Enterobacteriaceae and growth-negative specimens were excluded.

### 4.3. Identification of the Isolates

Bacterial isolates were identified by the routine techniques, which comprised cultural and morphological features and a battery of biochemical and motility tests. The species identification was further confirmed by API 20E (BioMérieux, Lyon, France). The antimicrobial susceptibility testing (AST) was determined by the disc diffusion technique following the CLSI recommendations and protocol [12]. Colistin susceptibility testing was performed by the broth microdilution method [12]. The quality control strains included *E. coli* (ATCC 25922) for AST and *Pseudomonas aeruginosa* (ATCC 27853) for carbapenem resistance. The break points for imipenem (IPM) and meropenem (MEM) against Enterobacteriaceae were interpreted as sensitive, intermediate and resistant at the MICs of ≤1 μg mL^−1^, 2 μg mL^−1^ and ≥4 μg mL^−1^, respectively, as recommended by the CLSI [12]. The MICs were performed by the Etest strip (BioMérieux, Lyon, France). The Rapedic CARBA NP (RCNP) kit was used for the phenotypic detection of carbapenemases. The test was performed according to the manufacturer’s instructions and standard operating procedures (BioMérieux, Lyon, France). The metallo-β-lactamases were phenotypically screened and detected by the double disc diffusion method using EDTA as a metallo-β-lactamase inhibitor and imipenem (IPM, 10 µg) and meropenem (MEM, 10 µg) disc, (Oxoid, Basingstoke, Hampshire, UK). After lawn formation on Muller Hinton agar (MHA) surface, IPM and MEM discs were placed 30 mm apart from one another, and a filter paper disc having 10 μL of 0.5 M EDTA solution was placed at the center of both discs. Inoculated plates were incubated at 37 °C overnight [21]. The synergistic effect of EDTA and carbapenems against metallo-β-lactamase producers appeared when the zone of inhibition due to the carbapenem discs increased with EDTA-containing discs.

### 4.4. Manual PCR Method

The RCNP positive isolates were selected to analyze metallo-β-lactamase production by targeting common genes, including *bla_VIM_* (F-GGTGTTTGGTCGCATATCGC R-CCATTCAGCCAGATCGGCATC), *bla_NDM_* (F-CACCTCATGTTTGAATTCGCC R-CTCTGTCACATCGAAATCGC) and *bla_IPM_* (F-GGAATAGAGTGGCTTAATTC R-CAACCAGTTTTGCCTTACC), with 503bp, 984bp and 327bp, respectively. PCR reaction (25 µL) was prepared in according to the master mix protocol (Promega, Madison, WI, USA). The PCR conditions were maintained as previously described [19,22].

### 4.5. Plasmid Extraction and Conjugation

The GeneJET plasmid miniprep kit (Thermo Scientific™, Waltham, MA, USA, #K0502) was used for the plasmid extraction, according to the manufacturer’s instructions and the protocol for amplification of *bla_VIM_*, *bla_IMP_* and *bla_NDM_*. The PCR mixture and conditions were the same as for the whole DNA sample amplification. Conjugation was performed as described by Borgia et al. [23]. The PCR positive *bla_NDM_* CRE strains were used as the donors with the recipient *E. coli* J53 (a sodium azide resistant) strain. The transconjugants were selected by inoculating a mixture of donor and recipient fresh cultures on Mueller-Hinton (MH) agar containing sodium azide (100 µg mL^−1^) and imipenem (1 µg mL^−1^). The *bla_NDM_* gene was detected in transconjugants by using PCR, with the same primers and conditions used for the whole genome DNA and plasmid of clinical isolates.

## 5. Conclusions

In conclusion, this study supports the dominance of NDM in this setting and supports continuous monitoring to control outbreaks and infection mitigating measures against the spread of these bugs.

## Figures and Tables

**Table 1:** Proportion of different species of Enterobacteriaceae (*n* = 238).

Species	Number (%) of CR *	Number (%) of CS **	Total No. of Isolates (%)
*E. coli*	18 (21.42)	66 (78.57)	84 (35.29)
*K. pneumoniae*	20 (28.98)	49 (71.01)	69 (28.99)
*Enterobacter cloacae*	9 (22.50)	31 (77.50)	40 (16.80)
*Klebsiella aerogenes*	3 (23.07)	10 (76.92)	13 (5.46)
*S. typhi*	-	10 (100)	10 (4.20)
*P. mirabilis*	2 (20.0)	8 (80.00)	10 (4.20)
*K. oxytoca*	-	6 (100)	6 (2.52)
*P. vulgaris*	-	4 (100)	4 (1.68)
*S. marcescens*	-	2 (100)	2 (0.84)
Total	52 (21.84)	186 (78.15)	238 (100)

* CR, carbapenem-resistant isolate (resistant to both imipenem and meropenem); ** CS, carbapenem susceptible.

**Table 2:** Phenotypic detection of carbapenemases by Rapedic CARBA NP test, MBL detection by EDTA synergy with carbapenems (double disc diffusion test, (DDST)) and carbapenemases by PCR in CRE isolates (*n* = 52).

Carbapenem-Resistant (CR) Species	Rapedic CARBA NP Positive No. (%), *n* = 52	MBL Positive No. (%), *n* = 52	PCR *bla_NDM_* No. (%), *n* = 52
*E. coli*	16 (30.76)	15 (28.84)	15 (28.84)
*K. pneumoniae*	19 (36.53)	17 (32.69)	16 (30.76)
*E. cloacae*	8 (15.38)	8 (15.38)	7 (13.46)
*Klebsiella aerogenes*	3 (5.76)	3 (5.76)	3 (5.76)
Total No. (%)	46 (88.46)	43 (82.69)	41 (78.84)

## Data Availability

Data associated with this work are given in the manuscript. Raw data can be obtained from the corresponding author upon a reasonable request.
